# Scoping Review on Active Teaching and Learning Methodologies in Dentistry

**DOI:** 10.1111/eje.13109

**Published:** 2025-05-07

**Authors:** Carlota Rocha de Oliveira, Andressa da Silva Barboza, Juliana Silva Ribeiro de Andrade, Rafael Guerra Lund

**Affiliations:** ^1^ Graduate Program in Dentistry, School of Dentistry Federal University of Pelotas Pelotas RS Brazil; ^2^ Graduate Program in Dentistry Federal University of Santa Catarina Florianópolis SC Brazil

**Keywords:** active learning, dental education, flipped classroom, gamification, problem‐based learning, undergraduate education

## Abstract

**Objective:**

The objective of this scoping review is to identify the most extensively researched active teaching and learning methodologies in undergraduate dentistry courses and to evaluate their potential benefits for enhancing student knowledge.

**Methods:**

The comprehensive review followed the guidelines proposed by PRISMA‐ScR. A search strategy was conducted in six electronic databases (Pubmed, Embase, Scopus, Web of Science, Visual Health Library (VHL), and Google Scholar) until December 2024. Two independent reviewers conducted the search, screening and data extraction. Eligible studies included clinical trials and observational studies, focusing on active methodologies in higher education, excluding review studies, case series and case reports. No restrictions were placed on the publication date or language. Risk of bias assessment used the ROBINS‐I tool for non‐randomised studies and RoB 2 for randomised studies, following PRISMA extension and AMSTAR 2 protocols.

**Results:**

The initial search yielded 10 999 studies. After reviewing the full texts, 36 studies were included out of the 48 initially identified. Most studies were non‐randomised. While they did not indicate significant differences between active and traditional learning, they highlighted the potential advantages of active methodologies. Problem‐based learning was the most investigated, followed by the flipped classroom and gamification.

**Conclusions:**

These methodologies offer potential benefits for undergraduate dental education, but this scoping review does not definitively establish their superiority over traditional teaching methods. Consequently, further well‐designed studies are necessary to validate the actual benefits of these methods in the teaching and learning process for undergraduate dental students.

## 1 Introduction

In recent years, the teaching and learning process in various fields has evolved to meet the diverse needs of learners and adapt to the rapidly changing landscape of education. As a result, new educational tools have gained momentum, receiving increased attention and application across different educational domains [1, 2, 3]. Additionally, the demand for health professionals in the job market has shifted toward individuals who not only reproduce information but also possess the ability to generate and contribute to knowledge throughout their professional journey. This changing landscape has transformed the traditional dynamics of teaching and learning [3, 4, 5].

In this context, active methodologies have emerged as potential catalysts for curiosity and students' engagement, enabling them to explore theories and novel concepts that may not have been covered in the classroom or previously considered by instructors. As a consequence, students became active contributors, fostering a sense of involvement and competence [2, 6, 7]. Several active teaching and learning methodologies have been identified for dentistry courses, including gamification [8, 9, 10, 11, 12], problem‐based learning (PBL) [5, 13, 14, 15, 16, 17, 18], flipped classroom [19, 20, 21, 22, 23] and storytelling [24, 25]. These methods need to be tailored to the specific realities and needs of students in order to effectively enhance the learning process.

Many review studies show active learning is well‐received and effective in dental education. However, reviews lack a comprehensive overview, focusing narrowly on specific strategies. They also fail to differentiate research across educational settings [26, 27, 28, 29, 30, 31]. Primary and review studies have found that active learning can be more effective than traditional lecture‐based teaching in dental education. However, review studies, in particular, have failed to provide a comprehensive view of the existing literature on active learning in dental education. This setting is uniquely important as it provides the foundational knowledge expected to be applied by students in laboratory and clinical environments. Given the proposed advantages of active teaching and learning methodologies, conducting a scoping review becomes a comprehensive approach to examining the existing literature in this area. It aids in identifying the current state of knowledge, recognising research gaps and pinpointing potential areas for future investigation. Ultimately, a scoping review on active teaching and learning methods provides valuable insights into the effectiveness of these approaches in promoting student learning and engagement, thereby informing future research and practice in this field. Therefore, the objective of this study is to conduct a scoping review to identify the most researched active teaching and learning methodologies in undergraduate dentistry.

## 2 Methods

### 2.1 Protocol and Registration

The study protocol adhered to the Preferred Reporting Items for Systematic reviews and Meta‐Analyses for Scoping Reviews (PRISMA‐ScR) checklist [31]. Additionally, the development of the scoping review protocol followed best practice guidance and reporting items provided by Peters et al. [32] (Appendix S1) and was preregistered in the Open Science Framework (OSF). The research question was formulated following the PCC strategy, where P represents the population (undergraduate dental students), C signifies the concept (active teaching and learning methodologies in dental education) and C indicates the context (undergraduate level).

The research question that guided this study is as follows: “Do active teaching methodologies increase knowledge compared to traditional teaching methods of undergraduate dental students?”

### 2.2 Eligibility Criteria

The inclusion criteria for this study encompassed research involving undergraduate dental students and focused on the utilisation of active teaching and learning methodologies in dentistry courses at the undergraduate level (Table 1). Eligible studies included those employing clinical trials (including non‐randomised studies and randomised controlled trials [RCTs]), as well as observational studies (such as cross‐sectional, case‐control, cohort studies and ecological studies). Reviews, primary studies, case series or case reports, literature reviews and studies from distance education courses were excluded from consideration. No restrictions were imposed on the publication year or language of the studies included in the review. Articles published in languages other than English were identified and included when they met the eligibility criteria. To ensure accurate data extraction, non‐English articles were translated using professional translation services or bilingual reviewers, and the extracted data were subsequently verified by experts in the field.

**TABLE 1:** Inclusion and exclusion criteria.

Criterion	Inclusion criteria	Exclusion criteria
Population	Undergraduate dental students	Studies not involving undergraduate dental students
Study design	Clinical trials, observational studies (cross‐sectional, case–control, cohort studies, ecological studies)	Literature reviews, case reports, case series, distance education studies
Intervention	Active teaching and learning methodologies in dentistry	Studies not addressing active learning methodologies
Language	No language restrictions	None
Publication type	Peer‐reviewed journal articles	Conference abstracts, unpublished studies, grey literature

### 2.3 Information Sources

Two independent reviewers (CRO and ASB) conducted a literature search until December 2024, without imposing any date restrictions. Seven databases were selected for the search: PubMed (Medline), Scopus, The Cochrane Library, Embase, Web of Science, Virtual Health Library (VHL) and Google Scholar.

An electronic search was conducted using the following keywords: ‘problem‐based learning,’ ‘flipped classroom,’ ‘gamification,’ and ‘active learning,’ adapted from PubMed MeSH terms. The search strategy was developed based on PubMed MeSH terms (Medical Subject Headings) and adapted according to other databases when necessary. Appendix S2 provides a description of the search strategy implemented across all databases. Additionally, the references of the included articles were examined to identify other potentially relevant studies.

### 2.4 Selection Process

After retrieving the articles from the databases, they were imported into the Rayyan web app (Rayyan, Qatar) to remove duplicate removal and facilitate the assessment process. Two blinded and independent authors (CRO and ASB), who had been previously calibrated, evaluated the titles and abstracts of all documents based on the predetermined inclusion and exclusion criteria. The full texts of the studies were thoroughly examined to determine their compliance with the pre‐established criteria. Only articles that met all selection criteria were included. In instances of disagreement, consensus was reached through discussion involving a third reviewer with greater experience in the field (RGL).

### 2.5 Data Collection Process and Analysis

A qualitative and narrative synthesis was performed based on the data items extracted from the included studies, using a standardised Microsoft Office Excel spreadsheet (Microsoft Corporation, Redmond, WA, USA). Two independent reviewers (C.R.O. and A.S.B.) reviewed and summarised the key findings from each study. In cases where information was missing, the authors of the studies were contacted via e‐mail to retrieve the missing data. If no response was received within 2 weeks, a follow‐up email was sent. If no response was obtained within a month, the study was excluded from the review.

### 2.6 Data Items

The two reviewers (C.R.O. and A.S.B.) extracted the following data from each article, organising them in Microsoft Office Excel 2013 (Microsoft Corporation, Redmond, Washington, USA): author, year, methodology, objectives, primary results, course subject and semester, sex, average age, assessment instrument and duration, and self‐perception evaluation (Table 1).

### 2.7 Evaluation Criteria

The primary objective was to operationalise the concept of the “most investigated active teaching and learning methodology” by identifying and analysing the frequency and depth of research conducted on different active teaching and learning approaches in undergraduate dentistry courses and explore its potential benefits for student knowledge. Specifically, this study examined the presence of evaluations, utilised instruments, assessment periods, and whether follow‐ups were conducted on students over time to analyse the impact of active methodologies on student learning.

The potential benefits of active methodologies extend to fostering greater engagement and motivation for learning amongst students. Furthermore, they contribute to enhancing students' confidence in performing various clinical procedures, ultimately leading to improved performance in assessments. Additionally, active methodologies serve as catalysts for reflective thinking, encouraging students to critically analyse and evaluate their own learning processes and outcomes. These aspects collectively create a more dynamic and effective learning environment, promoting deeper understanding and retention of knowledge.

### 2.8 Critical Appraisal of Individual Evidence Sources

The study included a critical evaluation of individual evidence sources, although it is an optional step according to the PRISMA extension for scoping review (PRISMA‐ScR) guidelines. Two independent researchers assessed the risk of bias in the articles included in this scoping review using the ROBINS‐I tool for non‐randomised studies (Table 2) and RoB 2 for randomised studies (Table 3), following item 12 of the PRISMA extension guide for scoping review reports and subsection 9 of AMSTAR 2 [31].

**TABLE 2:** Demographic findings.

Authors and year	Institution and country	Methodology	Objectives	Control group	Teaching mode	Primary results	Subject	Semester	Total sample (*N*); gender: male (M); female (F)	Average age	Assessment instrument	Rating	Assessment period	Self‐perception evaluation
Rich et al. (2005 [16]	School of Dentistry—University of Southern California, USA	PBL (problem‐based learning)	To analyse the outcomes of the PBL curriculum in dentistry	Yes	In‐person	No difference in performance between PBL (*n* = 134) and traditional (*n* = 233) students	Preclinical and clinical periodontics	Third‐year	*N* = 367; Not specified	Not specified	Writing and practice	During	Not specified	Not specified
Richards and Inglehart (2006) [33]	Health Sciences—University of Michigan, USA	CBL (case‐based learning)	To examine the impact of case‐based teaching on dental students' perceptions	No	In‐person	Increased student appreciation for the complexity of patient care.	Interdisciplinary treatment planning seminar	Second‐year	*N* = 204; M = 101; F = 95; Not specified = 8	Not specified	Writing	Before and after	Not specified	Not specified
Kieser et al. (2008) [25]	Oral biology course—Otago University, New Zealand	Storytelling	To evaluate the effectiveness of storytelling in a dental clinical anatomy course	No	In‐person	Higher reflective learning tendencies among students.	Dental anatomy	Third‐year	*N* = 60; Not specified	Not specified	Writing	After	Not specified	Not specified
Pileggi and O'Neill (2008) [34]	University of Texas Health Science Center at Houston Dental Branch, USA	TBL	To enhance diagnostic skills using TBL and ARS	No	In‐person	Higher final exam scores and enhanced teamwork and critical thinking skills.	Preclinical endodontics	Second‐year	*N* = 64; Not specified	Not specified	Writing	Before and after	Not specified	Not specified
Vahed (2008) [8]	Durban University of Technology, South Africa	Gamification	To utilise board games in teaching Dental Morphology	No	In‐person	Positive impact on student attitudes and learning	Tooth morphology	First‐year	*N* = 52; Not specified	Not specified	Written and observational	After	Not specified	Encouraged reflection and additional reading
Moreno López et al. (2009) [17]	European University of Madrid (UEM), Spain	PBL	To analyse outcomes of PBL in fifth‐year dental students	Yes	In‐person	Higher grades and more time spent on group work and literature analysis	Special care in dentistry	Fifth‐year	*N* = 51; Not specified	Not specified	Writing	After	Not specified	Not specified
Amer et al. (2011) [9]	School of Dentistry—University of Iowa, USA	Gamification	To compare traditional methodology with interactive learning using a game	Yes	In‐person	No significant difference in knowledge or clinical skills; preference for game‐based learning	Preclinical unit	First‐year	*N* = 80; M = 49; F = 31	21–39 years	Writing and practice	Before and after	Not specified	Encouraged reflection and additional reading
Kavadella et al. (2012 [19]	School of Dentistry—University of Athens, Greece	Flipped classroom	To develop and implement a hybrid oral radiology course	Yes	Hybrid	Students highly valued the flipped classroom methodology, noting increased motivation, satisfaction, and improved competence in endodontic procedures	Oral radiology	Tenth‐semester (final year)	*N* = 47; M = 14; F = 33	Not specified	Writing	Before and after	Not specified	This method promotes autonomy in studying
Youssef et al. (2012) [14]	School of Medicine—Ain Shams University, Egypt	PBL	To validate the effectiveness of case study learning in physiology	No	In‐person	Second‐ and third‐year students demonstrated competence, while fourth‐year students could benefit from further preparation	Physiology	First‐year	*N* = 262; M = 98; F = 164	Not specified	Writing and presenting a case	Before and after	Not specified	Appreciated patient interactions, highlighting enhanced learning, confidence, and critical thinking
Hannig et al. (2013 [10]	Medical School—RWTH Aachen University, Germany	Gamification	To create and evaluate a self‐paced blended learning module for alginate mixing	Yes	Hybrid	No differences were found between groups regarding the management of emergencies or regular treatment.	Dental materials, prosthesis and, implantology	Second‐year	*N* = 55 M = 22 F = 33	20–23 years	Writing	Before and after	Not specified	Not specified
McKenzie (2013) [35]	School of Dentistry—University of Alabama at Birmingham, USA	CBL	To focus on the acquisition of professional knowledge and skills	No	In‐person	Active dynamics were found to enhance undergraduate performance.	Case‐based education course	Second‐year	*N* = 37; M = 19; F = 18	Not specified	Writing	Before and after	Not specified	Not specified
Ratzmann et al. (2013) [36]	University of Greifswald, Germany	PBL	To investigate student acceptance of PBL in the orthodontic curriculum	Alternating between PBL and Traditional	In‐person	There was a statistically significant increase in post‐test results compared to pre‐test scores.	Orthodontics	Fifth‐year	*N* = 34; M = 13; F = 21	25.8 years	Writing	After	Not specified	Students reported that the game was a valuable experience that helped them recall and apply knowledge while promoting teamwork
Vahed, et al. (2014 [37]	Durban University of Technology, South Africa	Gamification	To assess the pedagogical quality of the Dental Morphology board game	No	In‐person	Students in the study group outperformed those in the control group on all assessments	Tooth morphology	First‐year	*N* = 83; Not specified	Not specified	Writing	Before and after	Not specified	Not specified
Rimal et al. (2015) [18]	School of Dental Surgery—BPKIHS, Nepal	PBL	To introduce and evaluate the feasibility and challenges of PBL in undergraduate dentistry	No	In‐person	Storytelling improved memory, reflection, and key skills while enhancing learning, professional competence, and community building	Oral biology, oral pathology, oral medicine and radiology, orthodontics, and oral surgery	Second‐year	*N* = 37; Not specified	Not specified	Writing	Before and after	Not specified	Boosted public speaking confidence and improved communication, organisation, teamwork, and professionalism
El Tantawi et al. (2016) [38]	School of Dentistry—University of Dammam, Saudi Arabia	Gamification	To measure student satisfaction with gamification in an academic writing course	No	In‐person	No significant difference was observed between the traditional method and flipped classroom, with pre‐ and post‐test mean scores of 3.98 ± 1 and 3.61 ± 1, respectively	Academic writing	First‐year	*N* = 92; M = 47; F = 45	20 years	Writing	Before and after	Not specified	The flipped classroom was a new and compelling experience, but students felt they gained very little from this method
Bai et al. (2017) [39]	School of Stomatology—China Medical University, China	PBL	To compare clinical problem‐solving skills and collaboration in PBL and traditional groups	Yes	In‐person	The case‐based activity and operative procedures increased student comfort and bridged the theory‐practice gap	Surgery	Fourth‐year	*N* = 90; M = 48; F = 42	Not specified	Written, telephone interview	After and 3 years later	3 years later	Not specified
Samuelson et al. (2017 [40]	School of Dentistry—University of North Carolina at Chapel Hill, USA	CBL	To compare the acceptability and effectiveness of CBL versus traditional instruction	Yes	In‐person	Students who used VDC scored significantly higher on qualifying tests, indicating its potential for predicting higher scores in periodontics and endodontics	Preclinical removable prosthesis	Second‐year	*N* = 82; Not specified	Not specified	Online learning management system (Sakai)	During, after, and 6 months later	6 months	VDC was effective for education purposes
Shigli et al. (2017 [41]	Dental School and Hospital—Bharati Vidyapeeth Deemed University, India	CBL	To compare knowledge of hyperplastic tissues in patients with complete dentures before and after CBL	No	In‐person	The game contributed to dental public health education in both dental schools, improving participants' knowledge, skills, and engagement	Prosthesis	Fifth‐year	*N* = 45; M = 15; F = 30	22.54 ± 0.83 years	Writing	Before and after	Not specified	After using the game, students reported that it helped them recall, enhance, and apply knowledge
Zain‐Alabdeen (2017) [20]	School of Dentistry—Taibah University, Saudi Arabia	Flipped classroom	To investigate advantages and limitations of flipped classroom in oral radiology	No	Hybrid	Increased discussion, motivation, and use of technology	Oral radiology	Fourth‐year	*N* = 100; *N* = 50; F = 50	Not specified	Writing and group discussion	During and after	Not specified	Not specified
Al‐Madi et al. (2018) [5]	School of Dentistry—Princess Nourah Bint Abdulrahman University, Saudi Arabia	PBL	To evaluate knowledge and confidence in Head and Neck Anatomy using PBL	No	In‐person	Significant improvement in knowledge and self‐confidence	Head and Neck Anatomy	First‐year	*N* = 30; F = 30; M = 0	Not specified	Writing	Before, during and after	The third year was 15 months after the end of the course	Not specified
Galvão et al. (2018) [13]	University Center San Lucas	PBL	To compare performance in PBL with traditional education	Yes	In‐person	No significant difference in oral radiology learning	Oral radiology	Second‐year	*N* = 129; F = 90; M = 39	≤ 24 years > 24 years	Writing	After	Not specified	Not specified
Lee and Kim (2018) [21]	Harvard School of Dental Medicine, USA	Flipped classroom	To develop flipped classroom approach and investigate its effectiveness	No	In‐person	Increased satisfaction, engagement, and learning outcomes	Periodontics	Third‐year	*N* = 71; Not specified	Not specified	Writing	Before and after	Not specified	Increased satisfaction and engagement
Melo Junior et al. (2018) [42]	University of Pernambuco—Campus Arcoverde, Brazil	Flipped classroom	To evaluate the effectiveness of the flipped classroom as a teaching‐learning strategy for coronary opening content	No	In‐person	Students responded positively to the flipped classroom methodology, reporting high satisfaction, increased motivation to study endodontics, and improved competence in endodontic procedures.	Primary Care in Oral Health II	Not specified	*N* = 14; Not specified	Not specified	Writing	Before and after	Not specified	This method promotes autonomy in studying
Chang et al. (2019 [15]	National Yang‐Ming University, Taiwan	PBL	To assess the impact of integrating standardised patients with PBL on undergraduate learning	No	In‐person	Second‐ and third‐year students demonstrated competence, while fourth‐year students could benefit from further preparation	Not specified	Second‐, third‐, and fourth‐year	Second year (*n* = 45); third year (*n* = 141); fourth year (*n* = 127)	Not specified	Writing	Not specified	Not specified	Improved learning, increased confidence, and fostered critical thinking
Isherwood et al. (2019) [22]	University of Liverpool, UK	Flipped classroom	To prepare final‐year students for orthodontic emergencies by comparing the inverted classroom with the traditional approach	Yes	In‐person	No differences were found between groups regarding the management of orthodontic emergencies or regular orthodontic treatment	Orthodontics	Final‐year	*N* = 61; Not specified	Not informed	Writing	After	Not specified	Not specified
Martins et al. (2019) [43]	State University of Ponta Grossa, Brazil	Flipped classroom	To compare flipped classrooms with traditional teaching methods	Yes	In‐person	Active dynamics were found to enhance undergraduate performance.	Periodontics	First‐ and second‐ year	Not specified	20.2 ± 1.7 years	Writing	After	Not specified	
Aubeux et al. (2020) [12]	School of Dental Surgery—University of Nantes, France	Gamification	To evaluate the impact and perceived value of an endodontic‐themed game as assessed by fourth‐year dental students	No	In‐person	There was a statistically significant increase in post‐test results compared to pre‐test scores.	Endodontics	Fourth‐year	*N* = 18; Not specified	Not specified	Writing	Before and after	Not specified	A valuable experience that reinforced knowledge and promoted teamwork
Qutieshat et al. (2020) [44]	School of Dentistry—Jordan University of Science and Technology, Jordan	Flipped classroom	To assess the effectiveness of a learning model combined with a flipped classroom approach in a clinical dental education setting, based on student performance and perceptions	Yes	Hybrid	Students in the study group outperformed those in the control group on all assessments.	Conservative dentistry	Fourth‐year	Control group = 364 (2016–17 cohort); Study group = 253 (2017–18 cohort)	Not specified	Writing and online	During and after	Not specified	Not specified
Zijlstra‐Shaw and Jowett (2020) [24]	School of Clinical Dentistry—University of Sheffeld, UK	Storytelling	To enhance the understanding of the potential role of storytelling in dental student education	No	In‐person	Storytelling aided memory, reflection, teamwork, communication, and technical skills, enhancing learning, professional competence, and community building	Primary care internship	Fourth‐ and fifth‐year	*N* = 57; Not specified	Not specified	Writing	Final	Not specified	Increased confidence in public speaking and enhanced technical skills, communication, organisation, teamwork and, professionalism
Adel et al. (2021) [23]	Saudi Arabia	Flipped classroom	To evaluate the effect of the sudden implementation of a flipped classroom on the perception and performance of undergraduate dental students, utilising regular questionnaires	Yes	Hybrid	No significant difference was observed between the traditional method and flipped classroom, with pre‐ and post‐test mean scores of 3.98 ± 1 and 3.61 ± 1, respectively	Periodontics	Fourth‐ and fifth‐year	*N* = 76; M = 40; F = 36	Not specified	Writting	Before and after	Not specified	The flipped classroom was a new and compelling experience, but students felt they gained very little from this method
Chutinan et al. (2021) [45]	School of Medicine—Harvard University Office of Human Research Administration, USA	CBL (case‐based learning)	To evaluate the effectiveness of the case‐based activity by assessing student comfort levels in operative procedures (injury approaches)	No	In‐person	The case‐based activity and the performance of operative procedures in teaching practice increased student comfort levels and bridged the theory‐practice gap between preclinical and clinical experiences	Operative dentistry	Second‐year	*N* = 172; M = 82; F = 90	Not specified	Writing	After	Not specified	Not specified
Wu et al. (2021) [46]	School of Dental Medicine—Kaohsiung Medical University, Taiwan	Gamification	To evaluate the validity and effectiveness of Virtual Dental Clinic (VDC) as an educational tool for clinical skills and knowledge acquisition among dental students	No	In‐person	Students who used VDC scored significantly higher on qualifying tests, indicating its potential for predicting higher scores in periodontics and endodontics	Dental clinic internship	Fifth‐year	*N* = 34; M = 21; F = 13	Not specified	Writing	After	Not specified	VDC was effective for education purposes
Salian et al. (2022) [47]	Manipal College of Dental Sciences, Manipal Academy of Higher Education, India	Comparative analysis (Traditional vs. Live‐field teaching)	To compare understanding and retention of oral pathology concepts using traditional and live‐field teaching methods	Yes	In‐person	Static‐field teaching had slightly better scores overall, but live‐field teaching excelled in specific topics	Third‐year dental students	Third year	*N* = 98; Gender distribution not specified	Not specified	Customised tests based on histopathological concepts	After	Not specified	Not specified
Sipiyaruk et al. (2022) [11]	King's College London in the King's Kingdom and Mahidol University, Thailand	Gamification	To evaluate knowledge improvement after interacting with the game, investigate student interaction during game completion, and investigate student perceptions of the game	No	In‐person	The game contributed to dental public health education in both dental schools, improving participants' knowledge, skills, and engagement	Public health	Final year	*N* = 12; F = 7; M = 5	Not specified	Writing	Before and after	Not specified	After using the game, students reported that it helped them recall, enhance, and apply knowledge
Hossain et al. (2022) [48]	College of Dentistry—Najran University, Saudi Arabia	TBL	To compare learning outcomes of TBL and traditional methods	Yes	In‐person	TBL showed significantly higher performance (58.33%) compared to traditional learning (44.4%, *p* = 0.01)	Periodontology	Mixed levels (Various sessions)	*N* = 72; Gender distribution not specified	Not specified	Multiple‐choice questions (MCQ)	During	Excellente	Not included
Varghese et al. (2024) [49]	KLE Vishwanath Katti Institute of Dental Sciences, KLE Academy of Higher Education and Research, India	Randomised controlled trial (Error‐based learning vs. lecture‐based learning)	To evaluate the effectiveness of error‐based active learning compared to lecture‐based learning	Yes	In‐person	Error‐based active learning showed better knowledge retention and positive student feedback	Final‐year dental students	Final year	*N* = 74; M = 18; F = 56	21–64 years	Self‐designed validated questionnaire (Cronbach's α = 0.87)	During and after	Significant improvement in knowledge retention (*p* < 0.001)	Positive feedback on active learning approach

**TABLE 3:** Risk of bias of non‐randomised clinical trials.

Author, year	1.1 Bias due to confounding factors	1.2 Bias in participant selection	2.1 Bias in the classification of interventions	3.1 Bias due to deviations from intended interventions	3.2 Bias due to missing data	3.3 Bias in outcome measurement	3.4 Bias in the selection of reported results
Rich et al. (2005 [16]	High	High	High	Some concerns	High	High	High
Richards and Inglehart (2006) [33]	Some concerns	High	Some concerns	Some concerns	High	Some concerns	High
Kieser et al. (2008 [25]	Some concerns	High	Some concerns	Some concerns	High	Some concerns	High
Pileggi and O'Neil (2008) [34]	Some concerns	High	Some concerns	Some concerns	High	High	High
Vahed (2008) [8]	Some concerns	High	Some concerns	Low	High	Some concerns	High
Moreno López et al. (2009 [17]	Some concerns	High	Some concerns	Some concerns	High	Some concerns	High
Kavadella et al. (2012) [19]	Some concerns	High	Some concerns	Some concerns	High	Some concerns	High
Youssef et al. (2012 [14]	High	High	High	Some concerns	High	High	High
McKenzie (2013) [40]	Some concerns	High	Some concerns	Low	High	Some concerns	High
Ratzmann el al. (2013) [36]	High	High	High	Some concerns	High	High	High
Vahed et al. (2014 [35]	Some concerns	High	Some concerns	Low	High	Some concerns	High
Rimal et al. (2015 [18]	Some concerns	High	Some concerns	Some concerns	High	Some concerns	High
El Tantawi (2016) [38]	Some concerns	High	Some concerns	Low	High	Some concerns	Some concerns
Samuelson et al. (2017) [40]	High	High	Some concerns	Some concerns	High	Some concerns	High
Shigli et al. (2017 [41]	Some concerns	High	Some concerns	Some concerns	High	Some concerns	High
Zain‐Alabdeen (2017) [20]	Some concerns	High	Some concerns	Some concerns	High	Some concerns	High
Al‐Madi et al. (2018) [5]	Some concerns	High	Some concerns	Some concerns	High	Some concerns	High
Galvão et al. (2018 [13]	Some concerns	High	Some concerns	Some concerns	Some concerns	Some concerns	Some concerns
Lee and Kim (2018 [21]	Some concerns	High	Some concerns	Some concerns	High	Some concerns	High
Melo Junior et al. (2018) [45]	High	High	Some concerns	Some concerns	High	Some concerns	High
Chang et al. (2019 [15]	High	High	High	Some concerns	High	High	High
Martins (2019) [43]	High	High	High	High	High	High	High
Aubeux et al. (2020 [12]	Some concerns	High	Some concerns	Some concerns	High	Some concerns	Some concerns
Qutieshat et al. (2020 [44]	Some concerns	High	Some concerns	Some concerns	High	Some concerns	High
Zijlstra‐Shaw and Jowett (2020) [24]	High	High	Some concerns	Some concerns	High	Some concerns	High
Adel et al. (2021 [23]	High	High	Some concerns	Some concerns	High	Some concerns	High
Chutinan et al. (2021 [45]	Some concerns	High	Some concerns	Some concerns	High	Some concerns	High
Wu et al. (2021) [32]	Some concerns	High	Some concerns	Some concerns	High	Some concerns	High
Hossain et al. (2022 [48]	Some concerns	High	Some concerns	Some concerns	High	Some concerns	High
Salian et al. (2022 [47]	Low	Low	Low	Low	Low	Some concerns	High
Sipiyaruk et al. (2022 [11]	Some concerns	High	High	Some concerns	High	High	High

## 3 Results

### 3.1 Study Selection

**FIGURE 1:**
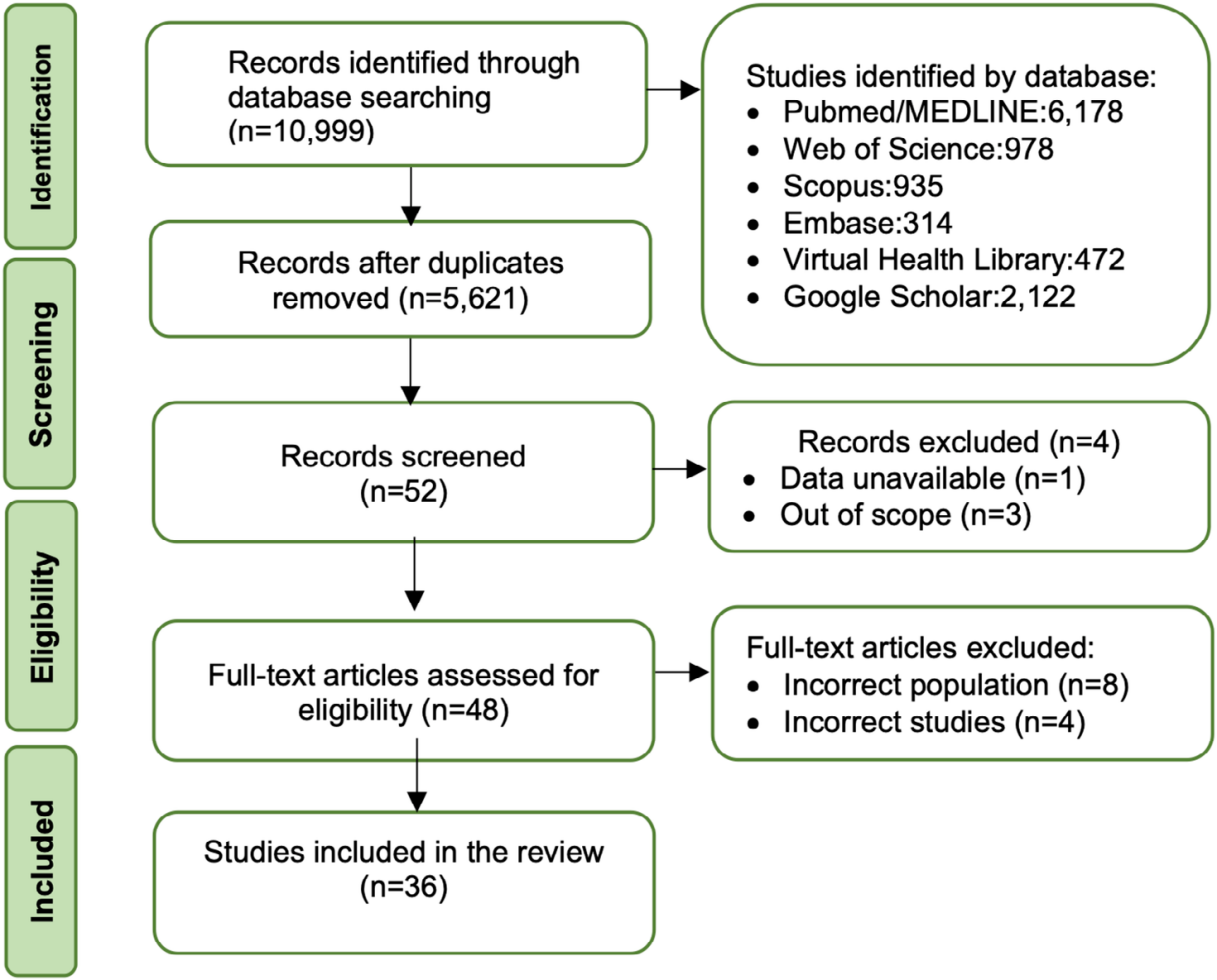
Flowchart depicting the PRISMA statement for database search results.

Initially, 10 999 studies were identified, and after removing duplicates, 5621 remained. The full texts of 48 studies were read, and 33 of them met the inclusion criteria. Additionally, three more studies were identified through manual search, bringing the total to 36 studies. Amongst the included studies, only five articles were randomised clinical trials and three articles were published in Portuguese. These articles were reviewed and translated by native Portuguese‐speaking researchers to ensure accuracy in data extraction (Figure 1).

### 3.2 Study Characteristics

Table 2 provides an overview of the main characteristics of the studies included in this scoping review. These studies explored various educational methodologies applied to different dental disciplines, encompassing traditional methods and innovative approaches such as PBL (*n* = 9), flipped classrooms (*n* = 8), gamification (*n* = 8), case‐based learning (CBL) (*n* = 7), storytelling (*n* = 2), error‐based learning (*n* = 1) and live‐field teaching (*n* = 1) [8, 9, 10, 11, 12, 13, 14, 15, 16, 17, 18, 19, 20, 21, 22, 23, 24, 25, 33, 34, 35, 36, 37, 38, 39, 40, 41, 42, 43, 44, 45, 47, 48, 49]. The samples primarily consisted of second‐ to fifth‐year dental students, with substantial variation in sample sizes, ranging from fewer than 20 to over 200 participants. For instance, Kieser et al. [25] involved 60 students, while Youssef et al. [14] included 262 participants. Gender distribution was explicitly reported in 18 studies, revealing diverse participant demographics.

Outcomes related to the effectiveness of these methodologies were mixed. Active approaches like PBL, CBL and gamification frequently demonstrated improvements in learning outcomes and student satisfaction. For example, Moreno López et al. (2009) [17] observed better group work engagement and grades in special care dentistry, whereas others, like Rich et al. [16] and Isherwood et al. [22], found no significant performance differences between traditional and active methods. Gamification and storytelling, though less frequently studied, showed promise. Aubeux et al. [12] noted significant score improvements in endodontics using gamification, while Vahed [8] highlighted positive attitudes fostered by a board game on dental morphology.

Flipped classrooms emerged as a widely adopted hybrid method, generally receiving positive feedback, with studies [20, 21, 22] reporting higher student satisfaction and competence. However, some findings, such as Adel et al. [23], revealed no significant differences in pre‐ and post‐test scores when comparing flipped classrooms to traditional methods. Overall, the evidence suggests that innovative methodologies can enhance academic performance and satisfaction, but outcomes depend on implementation and context.

### 3.3 Synthesis of Results

The synthesis of results underscores the varied impact of teaching methodologies on student performance and satisfaction. Gamification, PBL and flipped classrooms were particularly effective in enhancing motivation, fostering critical thinking and improving clinical competence in certain contexts. However, the outcomes varied depending on the subject matter and implementation. For instance, gamification often increased engagement but did not consistently lead to significant clinical improvements [8, 9, 10, 11, 12], while PBL [13, 14, 15, 16, 17, 18] and flipped classrooms [2, 19, 20, 21, 22, 23] frequently demonstrated better post‐test performance and knowledge retention. Similarly, storytelling enriched learning by encouraging reflection and improving communication, teamwork and technical skills [24, 25].

Despite these benefits, several studies found no significant differences in performance between active and traditional methods [36]. This suggests that factors such as course design and assessment timing play a crucial role in determining the effectiveness of these methodologies. Long‐term follow‐up studies provided inconclusive evidence on knowledge retention, highlighting the need for further research [5, 39, 40, 45].

### 3.4 Risk of Bias

Non‐randomised clinical trials usually had a high risk of bias or some concerns in the evaluated items (Table 3). On the other hand, randomised clinical trials demonstrated a low risk of bias in terms of intervention deviations (Table 4), but some concerns were noted in other domains.

**TABLE 4:** Risk of bias of randomised clinical trials.

Author, year	1. Randomization process	2. Deviations from intended interventions	3. Missing outcome data	4. Measurement of the outcome	5. Selection of the reported result	Overall risk of bias
Amer et al. (2011) [9]	Some concerns	Low	Some concerns	Some concerns	Some concerns	High
Hannig et al. (2013 [10]	Low	Low	Some concerns	Some concerns	Some concerns	High
Bai et al. (2017) [39]	Low	Low	Some concerns	Some concerns	Some concerns	High
Isherwood et al. (2019 [22]	Low	Low	Some concerns	Some concerns	Some concerns	High
Varghese et al. (2024 [49]	Low	Low	Low	Some concerns	Some concerns	High

## 4 Discussion

The main results of this scoping review identified PBL, flipped classrooms and gamification as the most investigated active teaching methodologies in undergraduate dental education. These methods showed potential benefits, including increased student engagement and motivation, enhanced confidence in clinical procedures and improved performance in assessments. Additionally, the review assessed the effectiveness of active methodologies in promoting student engagement, motivation and learning outcomes, and identified research gaps for future studies, such as the need for comparative studies of different active teaching and learning methodologies or exploring the role of technology in enhancing learning.

The included studies applied active teaching methodologies across various subjects for undergraduate dental students, such as curricular internships, preclinical units, dental materials, public health, physiology, endodontics, periodontics, anatomy, surgery, academic writing, dental anatomy, orthodontics, prosthesis and radiology. The results indicated a generally positive reception to these methodologies, particularly for fostering deeper engagement and reflective learning. However, contextual factors, including subject matter, course design and follow‐up duration, were critical in determining their effectiveness. The pre‐pandemic studies highlighted the existing value of self‐learning processes, while pandemic‐era studies demonstrated the adaptability of active methodologies to mitigate the impacts of social distancing on education. Most of the selected studies were conducted before the COVID‐19 pandemic, indicating that the self‐learning processes favoured by active learning methodologies were already valued in dental education. During the pandemic, studies utilised active methodologies to mitigate the negative impacts of social distancing on education, highlighting the continued need for tools that stimulate self‐learning [11, 23, 45].

The results of randomised clinical trials comparing active learning methodologies with traditional methods in dental education indicate a clear advantage for active approaches [9, 10, 22, 39, 49]. Studies have demonstrated that strategies such as error‐based learning, gamification, PBL and team‐based learning (TBL) result in better knowledge acquisition and retention compared to traditional lectures. For instance, error‐based learning proved superior in retaining knowledge about atraumatic restorative treatment, with positive feedback from students. Similarly, TBL not only enhanced learning outcomes but also encouraged self‐directed learning.

Additionally, methodologies like gamification and flipped classrooms have shown mixed results. Amer et al. [9] observed no significant difference in knowledge or clinical skills between traditional and game‐based learning in preclinical dental education, although students favoured the game‐based approach due to increased engagement and motivation. Hannig et al. [10] similarly reported no significant differences in practical outcomes with gamification but highlighted its potential to encourage reflection and additional reading. Bai et al. [39] demonstrated that PBL enhanced clinical problem‐solving skills and bridged the gap between theory and practice, particularly in operative procedures. However, Isherwood et al. [22] found no differences in managing orthodontic emergencies when comparing flipped classrooms to traditional methods. These findings collectively suggest that while active learning methodologies often improve knowledge retention and student satisfaction, their impact can vary based on the subject matter and implementation. Integrating these approaches into dental curricula is essential to optimise student learning experiences and outcomes. These mixed findings reinforce the importance of tailoring methodologies to the specific educational context to optimise their impact.

Contrary to expectations, most studies with control groups did not find significant differences in student performance between active methodologies and traditional teaching in clinical subjects [13, 16, 36, 39]. Only two studies reported that students felt more prepared and worked more constructively with PBL, yet no significant differences were observed [45, 50]. This highlights the variability in outcomes and underscores the need for further comparative research to identify factors influencing the success of these methodologies.

Flipped classrooms, extensively compared to traditional teaching, generally showed improved student performance in subjects like oral radiology, periodontics and conservative dentistry [19, 21, 42, 44]. Studies focusing solely on flipped classrooms without comparison also demonstrated improvements in subjects such as endodontics and anatomy [18, 42].

Gamification emerged as a beneficial tool for teaching and developing practical skills, enhancing student performance, knowledge and skills across various areas, including curricular internships, public health, academic writing, dental morphology and endodontics [8, 11, 12, 37, 38, 45]. Despite its overall positive reception, one study in an academic writing course noted students' reluctance to reuse gamification, likely due to the obligatory nature of participation and grade concerns [40]. Control group studies in subjects like preclinical units and dental materials found no significant performance differences, though gamification increased student motivation [9, 10].

CBL, often lacking control groups, showed performance improvements in prostheses and interdisciplinary treatment planning [33, 41]. Storytelling, used in primary care internships and dental anatomy, stimulated reflection, teamwork and memory improvement, though no comparative studies were found [24, 25, 42]. TBL was associated with performance improvements and critical thinking development [34].

Most courses assessed the effectiveness of these methodologies through written tests and self‐perception, with some using practical tests, case presentations, discussion groups and telephone interviews [9, 14, 16, 19, 39, 43]. Only one study used an online learning management system (Sakai) for assessments [40]. Most studies evaluated methodologies before and after application, aiming to identify their impact on students. However, the lack of follow‐up in most studies limits insights into the long‐term effectiveness of these methodologies. Future research should address this gap to determine their sustained impact. The literature search revealed four randomised clinical trials, but only one detailed the randomisation process [9, 10, 22, 39]. All studies had a high risk of bias, underscoring the need for well‐designed research. Improving study design, including the use of standardised outcome measures and larger sample sizes, will enhance the reliability of findings and their applicability to dental education.

It is important to note that many of the included studies exhibit variability in design, small sample sizes, and a lack of standardised outcome measures, which collectively limit the generalisability of the findings. Furthermore, publication bias may have influenced the results, as studies with positive outcomes are more likely to be published than those reporting null or negative findings. These limitations highlight the need for future research to adopt more rigorous methodologies, including randomised controlled trials with larger sample sizes and standardised evaluation protocols, to provide a clearer understanding of the effectiveness and long‐term impact of active teaching and learning methodologies in undergraduate dental education. While our study employed a rigorous methodological approach, it ultimately serves as a testament to the overall insufficiency of existing evidence to meet stringent quality criteria. Therefore, the central limitation lies in the inability to conclusively address our research inquiry due to these constraints. Moving forward, addressing these methodological shortcomings and establishing more rigorous standards will be imperative for advancing the field and providing clearer insights into the determinants of knowledge enhancement or learning efficacy, be it through testing, clinical outcome application, or oral health index control.

## 5 Conclusions

In conclusion, while this scoping review sheds light on the potential advantages of active methodologies in undergraduate dental education, it does not definitively establish their superiority over traditional teaching methods. The identified benefits encompass increased student engagement, enhanced confidence in clinical skills, improved academic performance, and the promotion of reflective learning. However, the lack of long‐term follow‐up in most studies underscores the need for further well‐designed research to ascertain the true efficacy of these methodologies in the teaching and learning process for undergraduate dental students. Future studies should aim to provide more robust evidence to guide educational practices in dental education.

## Author Contributions

All authors contributed to the study conception and design. Carlota Rocha de Oliveira: writing – original draft, writing – review and editing. Andressa da Silva Barboza: writing – original draft, writing – review, and editing. Juliana Silva Ribeiro de Andrade: supervision, writing – reviewand editing. Rafael Guerra Lund: project administration, resources, supervision, writing – review and editing. All authors have read and agreed to the published version of the manuscript.

## Conflicts of Interest

The authors declare no conflicts of interest.

## Supporting information


Appendix S1.

Appendix S2.

## Data Availability

The data presented in this study are openly available in Open Science Framework at https://doi.org/10.17605/OSF.IO/28ZVB.
