# Supplementary material for: Scoping Review on Active Teaching and Learning Methodologies in Dentistry
Source: Eur J Dent Educ. 2025 May 7;30(1):218–32. doi: 10.1111/eje.13109 (PMC12834545; doi:10.1111/eje.13109)
Supplement: Supplementary file 2 — Appendix S2. [file EJE-30-218-s002.docx]

| **Appendix 2. Supplementary data.** Comprehensive search strategy employed across all databases. | |
| --- | --- |
|  | ***PubMed (MedLine)*** |
|  | **Search terms** |
| **#1** | (Dental Education [Mesh] OR dental students OR student, Dental OR dental student OR dental educations OR education dentistry [Mesh] OR dentistry gamification OR dentistry Peer Instruction teaching) |
| **#2** | (problem based learning [Mesh] OR Learning, Problem-Based OR Problem Based Learning Curriculum OR Problem-Based OR Curriculum OR Problem Based OR Problem-Based Curriculum OR Problem-Based Curricula OR Curricula, Problem-Based OR Problem Based Curricula OR Experiential Learning OR PBL OR Active learning [Mesh] OR Learning, Experiential OR Learning, Active OR Inverted Sequence [Mesh] OR Inverted Sequences OR Sequence, Inverted OR Sequences OR Inverted Inversion OR Sequence Inversions OR Sequence Inversions OR games OR serious games OR simulation-game-based learning) |
| **#3** | (Clinical Trial OR Intervention Study OR Validation Study OR Evaluation Study OR Clinical Trials, Randomized OR Trials, Randomized Clinical OR Controlled Clinical Trials, Randomized)  #1 AND #2 AND #3 |
|  | ***Scopus*** |
|  | **Search terms** |
| **#1** | (“Dental Education” OR “dental students” OR “student, Dental” OR “dental student” OR “dental educations” OR “education dentistry” OR “dentistry gamification” OR “dentistry Peer Instruction teaching”) |
| **#2** | (“problem based learning” OR “Learning, Problem-Based” OR “Problem Based Learning Curriculum” OR “Problem-Based” OR “Curriculum” OR “Problem Based” OR “Problem-Based Curriculum” OR “Problem-Based Curricula” OR “Curricula, Problem-Based” OR “Problem Based Curricula” OR “Experiential Learning” OR “PBL” OR “Active learning” OR “Learning, Experiential” OR “Learning, Active” OR “Inverted Sequence” OR “Inverted Sequences” OR “Sequence, Inverted” OR “Sequences” OR “Inverted Inversion” OR “Sequence Inversions” OR “Sequence Inversions” OR “games” OR “serious games” OR “simulation-game-based learning”) |
| **#3** | (“Clinical Trial” OR “Intervention Study” OR “Validation Study” OR “Evaluation Study” OR “Clinical Trials, randomized” OR “Trials, Randomized Clinical” OR “Controlled Clinical Trials, Randomized”)  #1 AND #2 AND #3 FILTER: Dentistry |
|  | ***Web of Science*** |
|  | **Search terms** |
| **#1** | TS=(Dental Education OR dental students OR student, Dental OR dental student OR dental educations OR education dentistry OR dentistry gamification OR dentistry Peer Instruction teaching) |
| **#2** | TS=(problem based learning OR Learning, Problem-Based OR Problem Based Learning Curriculum OR Problem-Based OR Curriculum OR Problem Based OR Problem-Based Curriculum OR Problem-Based Curricula OR Curricula, Problem-Based OR Problem Based Curricula OR Experiential Learning OR PBL OR Active learning OR Learning, Experiential OR Learning, Active OR Inverted Sequence OR Inverted Sequences OR Sequence, Inverted OR Sequences OR Inverted Inversion OR Sequence Inversions OR Sequence Inversions OR games OR serious games OR simulation-game-based learning) TS=(Clinical Trial OR Intervention Study OR Validation Study OR Evaluation Study OR Clinical Trials, Randomized OR Trials, Randomized Clinical OR Controlled Clinical Trials, Randomized) |
| **#3** | TS=(Dental Education OR dental students OR student, Dental OR dental student OR dental educations OR education dentistry OR dentistry gamification OR dentistry Peer Instruction teaching)  #1 AND #2 AND #3 |
|  | ***Embase*** |
|  | **Search terms** |
| **#1** | (‘Dental Education’ OR ‘dental students’ OR ‘student, Dental’ OR ‘dental student’ OR ‘dental educations’ OR ‘education dentistry’ OR ‘dentistry gamification’ OR ‘dentistry Peer Instruction teaching’) |
| **#2** | (‘problem based learning’ OR ‘Learning, Problem-Based’ OR ‘Problem Based Learning Curriculum’ OR ‘Problem-Based’ OR ‘Curriculum’ OR ‘Problem Based’ OR ‘Problem-Based Curriculum’ OR ‘Problem-Based Curricula’ OR ‘Curricula, Problem-Based’ OR ‘Problem Based Curricula’ OR ‘Experiential Learning’ OR ‘PBL’ OR ‘Active learning’ OR ‘Learning, Experiential’ OR ‘Learning, Active’ OR ‘Inverted Sequence’ OR ‘Inverted Sequences’ OR ‘Sequence, Inverted’ OR ‘Sequences’ OR ‘Inverted Inversion’ OR ‘Sequence Inversions’ OR ‘Sequence Inversions’ OR ‘games’ OR ‘serious games’ OR ‘simulation-game-based learning’) |
| **#3** | (‘Clinical Trial’ OR ‘Intervention Study’ OR ‘Validation Study’ OR ‘Evaluation Study’ OR ‘Clinical Trials, Randomized’ OR ‘Trials, Randomized Clinical’ OR ‘Controlled Clinical Trials, Randomized’)  #1 AND #2 AND #3 |
|  | ***Web of Science*** |
|  | **Search terms** |
| **#1** | TS=(Dental Education OR dental students OR student, Dental OR dental student OR dental educations OR education dentistry OR dentistry gamification OR dentistry Peer Instruction teaching) |
| **#2** | TS=(problem based learning OR Learning, Problem-Based OR Problem Based Learning Curriculum OR Problem-Based OR Curriculum OR Problem Based OR Problem-Based Curriculum OR Problem-Based Curricula OR Curricula, Problem-Based OR Problem Based Curricula OR Experiential Learning OR PBL OR Active learning OR Learning, Experiential OR Learning, Active OR Inverted Sequence OR Inverted Sequences OR Sequence, Inverted OR Sequences OR Inverted Inversion OR Sequence Inversions OR Sequence Inversions OR games OR serious games OR simulation-game-based learning) TS=(Clinical Trial OR Intervention Study OR Validation Study OR Evaluation Study OR Clinical Trials, Randomized OR Trials, Randomized Clinical OR Controlled Clinical Trials, Randomized) |
| **#3** | TS=(Dental Education OR dental students OR student, Dental OR dental student OR dental educations OR education dentistry OR dentistry gamification OR dentistry Peer Instruction teaching)  #1 AND #2 AND #3 |
|  | ***Virtual Health Library (VHL)*** |
|  | **Search terms** |
| **#1** | (Estudantes de odontologia) OR (Students, dental) OR (Studiantes de odontología) OR (Étudiant dentisterie) OR (Dental Education) OR (dental students) OR (student, Dental) OR (dental educations) OR (education dentistry) OR (dentistry gamification) OR (dentistry Peer Instruction teaching) AND (Sala de aula invertida) OR (Gamificação) OR (Rotação por estações de aprendizagem) OR (Aprendizagem por Pares) OR (Aprendizagem baseadas em problemas) OR (Narrativas) OR (*storytelling)* |
| **#2** | (Ensaio clínico) OR (Clinical trial) OR (ensayo clínico) OR (essal clinique) OR (ensaio clínico controlado) OR (ensaio terapêutico) OR (ensayo clínico controlado) OR (Essai clinique contrôlé)  #1 AND #2 |
|  | ***Google Scholar*** |
|  | **Search terms** |
| **#1** | (Dental Education [Mesh] OR dental students OR student, Dental OR dental student OR dental educations OR education dentistry [Mesh] OR dentistry gamification OR dentistry Peer Instruction teaching) |
| **#2** | (problem based learning [Mesh] OR Learning, Problem-Based OR Problem Based Learning Curriculum OR Problem-Based OR Curriculum OR Problem Based OR Problem-Based Curriculum OR Problem-Based Curricula OR Curricula, Problem-Based OR Problem Based Curricula OR Experiential Learning OR PBL OR Active learning [Mesh] OR Learning, Experiential OR Learning, Active OR Inverted Sequence [Mesh] OR Inverted Sequences OR Sequence, Inverted OR Sequences OR Inverted Inversion OR Sequence Inversions OR Sequence Inversions OR games OR serious games OR simulation-game-based learning)  #1 AND #2 |
